# Stewed Fruit for the Gouty and the Dyspeptic

**Published:** 1883-08

**Authors:** 


### Stewed Fruit for the Gouty and the Dyspeptic.

Probably the impression first created by scanning the title of this paper will be as follows: "Why, what have either the gouty or the dyspeptic to do with stewed fruit at all?" That sugar is apt to disagree with sundry stomachs, causing great acidity, is a clinical fact not to be disputed. But because such is the case with a limited number of persons it does not seem, to me at least, that therefore a sweeping prohibitory law is to be laid down for a large section of the community. Gout poison, all admit, is a product derived from the albuminous constituents of our food, as nitrogen is a marked element in its composition. It is, then, the albuminous element in our food which has to be avoided in lithiasis. How sugar, fruits, and even vegetables came to be banned my researches have not enabled me to ascertain. From the time of Magendie's path-breaking essay writers on gout have advised the restriction of the nitrogenized, or azotized, constituents of our food in cases of lithiasis. But that the objection to sugar in gouty cases exists may not be denied. In speaking of stewed fruit for the gouty and the dyspeptic my views will be heterodox in the eyes of many. But neither the light of chemistry nor the lessons of practice conflict with my views. I am quite prepared to undergo any criticism these views may elicit or provoke. Indeed, the subject would be all the better for being thoroughly ventilated.

At the outset, I admit that for many persons—gouty, dyspeptic, and glycosuric—ordinary stewed fruit is objectionable from the amount of added sugar it contains. Where the acidity of fruit is masked or hidden by an excess of sugar, then the resultant product is cloying to many palates, and offensive to many stomachs. Probably in this all readers will agree with me. But it is by no means necessary to render stewed fruit objectionable by adding much sugar to it. Deprived of this excess of added sugar, stewed fruit can not only be rendered unobjectionable, but be converted into an actual prophylactic measure, especially in cases of lithiasis. In order to attain this end all that need be done is to neutralize the excessive acidity by an alkali, and then little or no sugar is required. Thrifty housewives have long been familiar with the fact that the addition of a small quantity of the bicarbonate of soda to stewed fruit reduced the acidity, so as to save the necessity for much sugar. This was done simply for economy. The principle has a far wider application. Last June I was requested to visit a lunatic in the Midlands who was also gouty; and when the gout was acutely present she was more excitable and violent than usual. No medicine would she take, but she was very fond of stewed fruit. To add potash to her stewed fruit was very easy, yet very effectual. After this I gave my cook instructions to perform a series of experiments for me with all our ordinary native fruits. The result of this was that the amount of bicarbonate of potash required for each pound of fruit was found to be about as much as would lie upon a shilling. And this is a much better guide for a cook than to put so many grains. With all fairly ripe fruit this was just sufficient to neutralize the acidity, and bring out the natural sweetness; indeed the resultant product was quite sweet enough for most adult palates. Such stewed fruit could be eaten alone, or with milk puddings, or with cream, or the Swiss milk in bottles. Gooseberries, currants of all kinds, apples and plums, all alike were excellent when so prepared. There are some points, however, to be attended to in practice, which are of more or less importance. The first matter is this: with dark fruits, as the black plum, for instance, the color is impaired by the alkali, and the fruit is less attractive to the eye than is that of the ordinary stewed fruit, which is of a deep clear crimson. This matter is easily got over; a little cochineal will give the desired color. Another is this: where there is no natural sweetness, to neutralize the acid completely by an alkali leaves nothing, simply a cold mass, to which the palate is absolutely indifferent. Such is the case with rhubarb. Here it is well to use half or all the amount of alkali with some sugar. The same is the case with early gooseberries before they have any natural sweetness, no sugar formed in them. Here the full quantity of alkali should be used, and the remaining acidity met by sugar. Where three-quarters of a pound of sugar is required to sweeten one pound of fruit, only one-quarter of a pound of sugar is necessary after the alkali has been added. The sour-sweet taste is thus secured, which is toothsome. Now, in these two instances the stewed fruit is only rendered less objectionable to the stomach plagued with acidity, not made quite inoffensive. But for ordinary gouty individuals not troubled with acidity of the stomach, such stewed fruit is quite admissible, and forms a pleasant method of taking potash. The whole subject is one which deserves attention from invalids as well as their medical attendants, as it opens up to many a new field of diet altogether. Fruits *au naturel*—as the strawberry, for instance—are good in gout from the salts they contain, and are unobjectionable stewed if it were not for the acetous fermentation of the added sugar.

Here soda may be used. But where there is lithiasis the alkali ought to be potash. The gouty and the bilious alike are troubled with the products of the metamorphosis of albuminoids. Neither the lithates of the gouty nor the bile acids of the bilious are derived from the saccharine or farinaceous elements of the food. It was possible to make bricks without straw, but it is impossible to make bricks without clay! No liver can make these nitrogenized substances from simple hydrocarbons; it is physically impossible! The dietary for each is the same—a non-nitrogenized dietary, in which vegetables and farinaceous matters are indicated, and saccharine matters, too, unless acidity in the stomach is produced by them. Milk puddings and stewed fruit are excellent for the dyspeptic, the bilious and the gouty, as my experience tells me; and for one of those who suffers from taking sugar, nineteen would be all the better for stewed fruit. But for those who dislike sweets, and for those who suffer from acidity, it is well to prepare the stewed fruit with alkalies, completely and solely or partially, as the case may be. This may sound very heterodox to some readers; but just let the incredulous test the matter for themselves.

Now, there are two other matters remaining to be alluded to, on which it is impossible to speak dogmatically, or *ex cathedra;* they are, rather, matters of personal belief, and they are these: 1. It does not seem a matter of indifference in lithiasis what forms of albuminoids are taken. The flesh of animals is rather converted into peptones by pepsin in an acid medium—that is, by gastric digestion—than by trypsin in an alkaline medium. And such peptones seem specially liable to form lithates. Caseine is more specially digested by trypsin in the intestine, and such caseine peptones seem less readily converted into lithates; the clinical fact being that a milk dietary or a pulse dietary is good for the subjects of lithiasis. Caseine is the form of albuminoid, it seems to me, best suited to the gouty. Milk or milk puddings (made without eggs) are capital food for the cholæmic or the lithæmie. These fibrin-albuminoids, digested by pepsin, are laxative, while casein is binding or constipating.

Milk puddings, then, go well with stewed fruit, which is a laxative. Many thoughtful physicians agree with me in the above matter. Now I am approaching what some may hold very disputable ground, yet nevertheless I venture to say here what I am beginning to think. Both for the classical diabetic and the glycosuric, cane sugar—the sugar of commerce—is bad, producing the unpleasant symptoms of sugar in the blood very readily. Yet many glycosuric individuals can take farinaceous matter with comparative immunity from discomfort. Starch in its way to grape sugar is much less troublesome than in cane sugar passing into grape sugar; why I do not know, but the fact remains. Now, with many glycosuric individuals fruit stewed in the manner advocated here is quite permissible, while ordinary stewed fruit is very objectionable. For the dyspeptic, the gouty, the bilious and the glycosuric individual (as well as the rheumatic, a small class), fruit stewed with an alkali in the proportion of as much bicarbonate of soda as will lie upon a shilling to the pound of fruit, when put in the oven, will be found both palatable and permissible. It saves the gastric acidity from the acetous fermentation of the sugar in the dyspeptic, or with the glycosuric relieves him from the excess of cane sugar which disagrees with him. Where there is distinct gout not only is fruit stewed with an alkali good and unobjectionable; but if it be prepared with the bicarbonate of potash, it is converted into a therapeutic agent of no mean value; while the resultant product is quite sweet enough for a palate which has outlived the "sweet-tooth" period. The whole matter is a simple one, yet it seems to contain much promise for many persons.—*J. Milner Fothergill in The Lancet.*

